# English language proficiency, perception and competence among the academic staff of public universities in Kosovo

**DOI:** 10.12688/openreseurope.19958.2

**Published:** 2025-10-07

**Authors:** Sejdi Sejdiu, Rezarta Ramadani, Aferdita Ninaj

**Affiliations:** 1 Department of English Language and Literature, University "Ukshin Hoti" Prizren, Prizren, Kosovo, 2000, Kosovo (Serbia and Montenegro)

**Keywords:** English, proficiency, perception, competence, academic staff, higher education, Kosovo

## Abstract

**Background:**

English language proficiency is increasingly vital for higher education institutions due to globalization and the internationalization of academic programs. In Kosovo, public universities are experiencing similar pressures to adopt English as a medium of communication and instruction.

**Methods:**

This study investigates how academic staff perceive their English language proficiency and its influence on their academic responsibilities and institutional roles. A mixed-method case study was conducted involving 336 academic staff members from all public universities in Kosovo. Data were collected through an online questionnaire distributed via email between October and December 2024. The survey included both closed- and open-ended questions assessing self-perceived language proficiency, usage in professional contexts, and attitudes toward English in higher education.

**Results:**

Revealed that most lecturers rated their English proficiency as “good” or “very good,” and a majority acknowledged the importance of English for academic work, publication, and international collaboration. While English was not a formal job requirement for many, respondents agreed that it facilitates research, access to academic resources, and professional networking. Key challenges included lack of formal training and limited institutional support. Open-ended responses emphasized the role of English in improving academic quality, access to international literature, and communication with foreign colleagues. English language proficiency plays a crucial role in the academic and professional development of university staff in Kosovo. Despite institutional gaps in formal language policies, the increasing demand for English in research, teaching, and international engagement signals the need for strategic support in language development.

**Conclusion:**

This study highlights the urgency of aligning institutional goals with language competence to ensure quality and competitiveness in the global academic arena.

## Plain Language Summary

This study looks at how well university lecturers in Kosovo believe they know English, how they use it in their work, and why it matters for their careers and institutions. English is increasingly important in higher education because it allows access to international research, supports teaching quality, and enables collaboration with colleagues abroad.

The research involved 336 lecturers from all seven public universities in Kosovo. They completed an online survey about their English skills, how they use English at work, and their opinions on the role of English in academia. Most lecturers said their English proficiency was “good” or “very good,” especially in reading and writing. Many agreed that English helps them publish research, use technology, and participate in international projects. They also noted that knowing English has expanded their professional networks and opened new career opportunities.

At the same time, the study found challenges. Not all universities require English proficiency as a condition of employment, and many lecturers have not received formal training or institutional support to improve their skills. Some also reported that limited English knowledge made it harder to engage with international publications and colleagues.

The results suggest that while Kosovo's academic staff recognize the importance of English, universities need clearer policies, training opportunities, and internationalization strategies to support staff language development. Improving English proficiency is not only valuable for individual lecturers, but also essential for making Kosovo's universities more competitive and better connected in the global academic community.

## Introduction

**‘English, the global language’ is a phrase often articulated in everyday discourse.**

In recent years, the English language is something you hear from politicians all across the world, say on the news. English advertising and billboards can be found almost everywhere; however, this does not mean everyone speaks English ^1^. There are also countries around the world that do not recognize English as a global language. But what makes English a global language? Firstly, a language can be proclaimed as the dominant language of a country, allowing it to be used as a communication medium in areas such as politics, the judiciary, the press, and academia, and frequently, that language is referred to as a ‘second language’. Secondly, even if a language seems to have no formal position, it can also be prioritized in a country's foreign-language teaching. Global academies as well as commercial communities recognize the need for a global language, and it is here that perhaps the acceptance of a common global language is most evident, both in lecture halls, and conference rooms, and also in the hundreds of individual connections made every day all over the world. What makes it special, is that when it comes to education, English, as a lingua franca, provides access to knowledge and serves as a unifying thread, while also opening doors to a global exchange of ideas ^2^.

As mentioned above, the era in which we find ourselves now is unanimously associated with globalization, where the need for a common language is felt more deeply and strongly than before. Clearly, that language is none other than English, with an unprecedented extension worldwide and in many domains. English does the job of bridging people with whom they have nothing in common. Out of the domains where English is widely spoken, it is certainly in the sector of higher education. In this context, English has been the language of Higher Education Institutions (HEI), in particular in those universities that are directed abroad for the establishment of international relations ^3^. Of course, many higher education institutions, also through the effects of the faster process of internationalization, have assumed many strategies to meet the demands of the internationalization of higher education, which is described as “the process of integrating an international/intercultural dimension into teaching, research, and service functions of the institution.” The most notable strategy pursued by universities to achieve 'Internationalization' was to change English as the language of instruction, either fully or partially on campus ^4^.

The pressure on universities to improve their global profile in the competitive higher education sector has made internationalization more important for higher education institutions today. Universities look beyond national borders. University councils set goals and devise strategies for internationalization. Some of these strategies have been agreed upon at the national and international levels, such as the Bologna Declaration of 1999 ^5– 8^. Since Bologna, the 29 signatory states have been implementing the Bachelor-Master-Doctorate structure and are implementing other instruments (ECTS (European Credit Transfer System) credits, diploma supplements, etc.) that are intended to pave the way for higher education. In addition, a European Higher Education Area has been created that is internationally competitive and attracts students from inside and outside Europe.

These trends highlight the issue of the European language and the challenges of communication in a multilingual environment. They force European higher education politicians to face the language issue and think about converting their curricula from the national language to the international standard - the English language. However, this process is not without its problems, especially since lecturers may have language restrictions. There may also be obstacles at other levels due to the cultural and political sensitivity of the issue. For example, policymakers at the local level may feel that the switch to English will come at the expense of the local language and teaching traditions, or there may even be legal restrictions on adopting such a policy ^9^.

Not only is the Bologna Process seen as vital in the internationalization process, but Erasmus as well. ERASMUS (European Region Action Scheme for the Mobility of University Students) was founded in 1987 with main goals such as training youth, supporting personal and professional development of students in education, in Europe and other countries elsewhere. Erasmus+ has played a pivotal part in a more comprehensive and deliberate approach to the internationalization process of higher education institutions in Europe, and has served as a model for universities, countries, and regions around the world. The impact is quite extensive, or, more accurately, Erasmus+ in the 1980s served as a catalyst for a more deliberate approach to the internationalization of higher education institutions. Erasmus+ laid the groundwork for the Bologna Process' inception and built mechanisms to facilitate it, such as the European Credit Transfer System (ECTS) ^10^. When it comes to mobility, the focus is usually on students. While many higher education institutions in Europe have procedures in place to increase student exchanges, there is seldom a deliberate effort to encourage staff mobility, despite the fact that the Erasmus+ program provides financing for academics including administrative personnel ^11^. A much more systematic approach to academic mobility has obvious benefits in terms of improving research, and education, as well as overall professional growth. Lecturers are critical to any educational institution's global expansion ^12^.

The Bologna Process and the HEIs have faced the issue of quality assurance, and there has also been a greater emphasis on the globalization of quality assurance, as well as how national mechanisms in Europe collaborate to produce common criteria and generally recognize their certification judgments. Accreditation departments are expected to look into the advancements and identify the most complicated matters associated with them as the HE (Higher Education) ideology is progressively globalized and cross-border proposals such as franchises, campuses, correspondence courses, joint and dual degree programs influence accreditation and quality assurance. Clearly, the internationalization process of higher education in Europe appears bright, but its influence will only be realized if different stakeholders and partners in this ongoing transition process embrace a new discussion regarding justifications, advantages, resources, possibilities, and challenges ^13^.

The aim of this study is to explore the English language proficiency, perceptions, and competence of academic staff across Kosovo’s public universities. It addresses the gap in existing literature, as no prior study has systematically investigated English proficiency and its role in internationalization in the Kosovar higher education context. The study contributes by providing empirical insights that can inform EMI policy, staff development, and internationalization strategies in Kosovo.

### Literature review

Guided by the gaps in previous research, this study is driven by the following research questions: (1) How do lecturers perceive their English language proficiency and competence? (2) Is English language proficiency seen as crucial for their work.

Known as the Latin of the 21st century, the English language is now playing the most powerful function in the internationalization of higher education. On a global scale, the English language is used mainly by 53 percent of the professionals in the CAP (Changing Academic Profession) survey; 17 percent use it as their native language and 36 percent use it as their second or third language ^13^. Despite the fact that 51 percent of academics use English as a lingua franca for research purposes rather than teaching purposes, only 30 percent use it for teaching. This disparity mostly affects non-native speakers. Almost all academics who are native English speakers utilize English for both teaching and research. While the use of the English language as a foreign language is a strong sign of the internationalization process of the universities in countries where English is not a dominant language, the link between the usage of the English language and the internationalization process of university is less evident in English-speaking nations. Academic institutions and professors in these nations have an advantage since practically all of them or most of the academics speak English. However, the usage of the English language as a language cannot be regarded as an indicator of engagement in worldwide research networks. Furthermore, the usage of the English language relates to academics and their contributions to the internationalization process of their higher education ^13^. 

Today, internationalization and globalization have a significant influence on higher education. This is because higher education is both an actor and a reactor in these processes. It is an actor in the sense that it is the agent of internationalization and globalization, and a reactor in the sense that it responds to the effects of internationalization and globalization. Regarding higher education, internationalization of higher education can be defined as the process of integration into the international/intercultural dimension in the teaching, research, and Institutional service functions ^14^.

While studies on EMI and faculty English proficiency are increasing in Western and Northern Europe (e.g., Denmark, Spain), there is still a striking absence of systematic research in the Western Balkans. This lack of empirical evidence is significant given the region’s participation in the Bologna Process and Erasmus+ initiatives, where English proficiency plays a crucial role in internationalization. Recent developments in EMI scholarship have further enriched the field: for example, Macaro and Aizawa (2024) ^15^ explore the interface between EMI and EAP/ESP research agendas, Huang & Fang (2023) ^16^ investigates how EMI teachers integrate cultural dimensions in practice, and Lee *et al.* (2025) ^17^ provide meta-analytic evidence of EMI’s positive impact on both content learning and language proficiency. Meanwhile, Wang, Yuan, and De Costa (2025) ^18^ critically review EMI teacher development across higher education. Despite these advances, no empirical study has yet examined academic staff’s English proficiency, perceptions, and competence in Kosovo’s higher education. Addressing this absence, the present study provides the first large-scale analysis of these issues across Kosovo’s public universities

If we look at universities more at the local level, it is found that, despite global pressure, reactions depend on their past and history ^19, 20^. Although internationalization is considered an indicator of success, a university will thrive once it has developed strong connections with local partners and agencies. This global-local dialectic becomes evident when we look at universities, which are global institutions rooted in local traditions and networks. Universities that have become entrepreneurial organizations have also been researched ^21^. They are characterized by a strong core of leadership; a periphery of development; a diversified financing base; a strong academic heart, and strong incentives for internationalization and transfer.

Universities may be right in their concerns about students' English ability. But what about the English abilities of faculty members? Do universities require them to demonstrate their English proficiency to determine if they are competent in teaching in English? At the time, a policy or practice did not appear to be under consideration in HE, at least in the form of a written or official declaration in white papers ^22^. Because there are no clear-cut, stated or unwritten standards regarding lecturers' English skills for employment, there is relatively little information in the literature on professors' attitudes toward their English skills and practices.

EMI (English Medium Instruction) describes the utilization of English to teach a non-linguistic course in a culture where English is not the dominant language and it has become one of the most significant trends confronting HEI in such situations today. The first wave of growth occurred in Europe, where most of the EMI study was undertaken. In Europe, there were nearly 11 times more EMI programs in 2014 than in 2001 ^23^. Linguistically, only a few academics have been involved in studies regarding the perceived English language competency of HE stakeholders and their use at EMI institutions. For example, in their study of the efficiency of the EMI policy in the Korean setting, discovered that instructors were dissatisfied with their English language abilities ^24^. A group of non-native English lecturers reported a variety of issues with their language usage and abilities in a study ^25^, which mostly covers problems linked to oral language output, such as pronunciation, accent, fluency, and intonation-related complaints. Similarly, the same issue was identified among 44 lecturers who were reported to have the most difficulty with pronunciation while teaching subject courses ^26^. It was discovered, however, that professors expressed a common concern about their language competence: being unable to cope with EMI linguistically at a desirable and desired level. Finally, in contrast, a research study discovered that lecturers were satisfied with their English skills and gave favorable self-evaluations ^27^.

On the other hand, over 45 percent of the academic staff at Leiden and Delft Universities in the Netherlands have a level of proficiency at C1 or higher ^28^, and this level is the minimum requirement for the university to teach in English at more than half of the 70 European universities surveyed ^29^.

Internationalization of higher education institutions in the Western Balkans is largely considered as a tool for assisting national changes and strengthening productive capacities ^30^. Numerous international organizations, particularly the European Union, provide economic support to most internationalization activities. Because the nations and systems in the Western Balkans are so different, it is hard to make broad generalizations. Higher education institutions and study are still poorly funded in many nations, universities are losing their best academic staff and students, who have already gone abroad, and foundational reforms are hard to implement due to a lack of ideological freewill and weak governance at both the systemic and institutional levels. Transnational proposals, such as the Bologna Process Communiques, frequently result in very minor modifications in national systems. Cross-functional and cross-collaboration offer tremendous potential for the internationalization process. There are already a number of key programs in place to promote collaboration in academic programs. According to the results of one opinion poll, academics in the nation are ready to collaborate with partners from Western Balkan institutions. When asked if their institution should prioritize collaboration with universities or higher education institutions in the Western Balkans, academics from Kosovo (98.3%) and Albania (93.5%) agreed with this statement, whereas those from North Macedonia (77.6%) and Bosnia and Herzegovina (60%). The other four countries that disagreed with this statement are: Montenegro (44.3%), Serbia (27.5%), Croatia (26.2%), and Slovenia (20.4%). Individual countries hunt for partners and form connections in the burgeoning European Higher Education Arena based on a sense of proximity and a shared tradition that they want to preserve and enhance ^30^.

Ultimately, the purpose of English nowadays in higher education is becoming more essential every day ^31^.

## Methodology of the study

### Methodology background

The purpose of this study was to explore the English language proficiency, perceptions, and competence among the academic staff of public universities in Kosovo, the internationalization of higher education in Kosovo, and the development of the English language as a global language in public universities in Kosovo. The study adopts a questionnaire-based cross-sectional survey design with a limited qualitative supplement (two open-ended questions) to analyze lecturers’ perceptions of English proficiency, its perceived advantages in university contexts, and its role in internationalization

### Participants

The data were collected in the setting of Kosovo Higher Education by surveying lecturers of the public universities of Kosovo. The participants were from the following universities: University “Hasan Prishtina” of Prishtina, University “Ukshin Hoti” of Prizren, University “Haxhi Zeka” of Peja, University “Kadri Zeka” of Gjilan, University “Isa Boletini” of Mitrovica, University “Fehmi Agani” of Gjakova, University of “Applied Sciences” of Ferizaj. Table 1 presents the demographic characteristics of the academic staff who participated in the study, including their gender, age group, and years of experience. The sampling of the research consisted of a total of 336 lecturers. The questionnaire link was distributed via email to academic staff across the seven public universities, and 336 lecturers completed it voluntarily. This approach represents a form of convenience sampling, since participation was based on voluntary self-selection rather than random assignment. Participation was voluntary, and written informed consent was obtained prior to completing the questionnaire. Ethical approval for the study was granted by the Scientific Committee of the University ‘Ukshin Hoti’ Prizren.

**Table 1.** Demographic characteristics of participants.

		Frequency (f)	Percentage (%)
Gender	Female	150	44,6
	Male	186	55,4
Age	25–30	32	9,5
	30–39	85	25,3
	40–49	104	31,0
	50–59	86	25,6
	60+	29	8,6
Years of teaching	0–10	124	36,9
	11–20	133	39,6
	21+	79	23,5

### Research design

This study employed a predominantly quantitative survey design, supplemented by qualitative insights from two open-ended questions. The main instrument was a structured questionnaire that gathered self-reported data on lecturers’ English proficiency, use of English in academic contexts, and attitudes toward its role in higher education. Quantitative items were analyzed descriptively and presented in percentages and charts, while responses to the open-ended questions were examined thematically to capture lecturers’ perspectives in greater depth. This design allowed for both statistical overview and richer contextual understanding of how English proficiency is perceived and experienced by academic staff in Kosovo’s public universities

### Instruments and Data Analysis

The questionnaire was designed as a spreadsheet to collect data and consisted of 20 questions addressing the main objectives of the research, including yes/no questions, multiple-choice questions, and two open-ended questions. Responses to the open-ended items were analyzed thematically: recurring patterns were identified and grouped into themes (e.g., access to international literature, career opportunities, communication with colleagues), and representative quotations were extracted to illustrate key points. Quantitative responses were analyzed descriptively, with results displayed in pie charts showing the distribution of answers as percentages. The survey on language perception and proficiency was conducted from October to December 2024. Overall, the data indicated that most lecturers held a positive view of their English language proficiency and its use in their academic work. In order to ensure the robustness of the questionnaire as a data-gathering instrument, steps were also taken to establish its validity and reliability, as outlined below. 

### Instrument Validity and Reliability

The questionnaire was designed after reviewing prior studies on English proficiency and EMI in higher education (e.g., Byun *et al.*, 2010 ^6^; Dearden, 2015 ^12^; Karakaş, 2014 ^26^) to ensure that the items were conceptually aligned with established constructs. To strengthen content validity, two senior academics in applied linguistics and language education reviewed the instrument for clarity, relevance, and coverage of the research objectives. Their feedback led to minor adjustments in the wording of several questions. In addition, the instrument was piloted with a small group of 12 academic staff members from two public universities in Kosovo. The pilot confirmed that the instructions and items were clear and comprehensible, and no major revisions were required.

While formal statistical reliability testing (e.g., Cronbach’s alpha) was not conducted, internal consistency was addressed through careful construction of thematically related clusters of items (e.g., self-assessment of proficiency, perceived importance of English, use of English in academic tasks). The similarity of responses within these clusters during piloting, as well as the expert review process, provides reasonable assurance of the instrument’s reliability. The similarity of responses within these clusters during piloting, as well as the expert review process, provides reasonable assurance of the instrument’s reliability.

It should be noted that the self-assessment of English proficiency relied on general labels (e.g., “basic,” “good,” “very good”) rather than standardized descriptors such as the Common European Framework of Reference (CEFR). This may limit the comparability of responses across studies and the precision of the proficiency levels reported.

### Limitations of the method

As the study relied on self-reported proficiency, results may reflect perceptions rather than tested language competence. In addition, the study relied solely on descriptive statistics; more advanced analyses such as correlations, regression models, or ANOVA were not performed. Future research could apply these methods to explore relationships between demographic factors (e.g., age, academic title) and English proficiency.

## Results

The following table presents the summarized responses from the questionnaire conducted among academic staff in Kosovo's public universities regarding English language proficiency, perception, and competence.

Analysis of Results:

1. Mastery of English Language

As shown in Table 2, the majority of respondents rated their English language proficiency as either ‘good’ or ‘very good’, particularly in reading and writing skills.

**Table 2.** Results excerpted from the questionnaire.

Question	Results
How well do you master the English language?	Good (39.2%), Very good (35.0%), Excellent (12.6%), Sufficient (9.9%), Poor (3.3%)
Has your English proficiency helped you land the job you currently have?	Yes (61.3%), No (38.7%)
Was English language competence a requirement for your job?	Yes (38.4%), No (61.6%)
I think knowledge of the English language is necessary for the job I do.	Agree (39.3%), Strongly agree (56.5%), Disagree (3.3%), Strongly disagree (0.9%)
Have you faced any situations when your lack of English proficiency was an obstacle for your work?	Yes (181), No (155)
I believe knowledge of the English language improves performance in other academic areas.	Strongly agree (62.8%), Agree (34.5%), Disagree (2.4%), Strongly disagree (0.3%)
My English proficiency has helped me in such cases as when particular materials in my mother tongue were hard or impossible to find.	Strongly agree (58.9%), Agree (37.2%), Disagree (3.6%), Strongly disagree (0.3%)
University staff must be proficient in another language other than their mother tongue.	Strongly agree (69.9%), Agree (30.1%), Disagree (0.3%)
How much does not knowing the English language hinder the development or implementation of key university components?	A little (5.7%), Considerable (38.1%), A lot (56.3%)
In which languages do you publish your scientific articles?	Albanian (16.7%), English (72.9%), German (2.1%), Albanian & English (2.7%), Turkish & English (1.5%), Albanian & German (0.6%), English & German (0.6%), Other (3.0%)
Technology requires some type of English proficiency.	Strongly agree (64.3%), Agree (33.0%), Disagree (2.7%)
Knowing English has helped me double my professional network, leading to additional opportunities.	Strongly agree (67.0%), Agree (29.2%), Disagree (3.9%)
Evaluate your English language abilities (1–10 scale).	1 (5), 2 (5), 3 (8), 4 (19), 5 (35), 6 (69), 7 (90), 8 (56), 9 (25), 10 (24)
How much do you believe the English language helps you in your academic staff? (1–10 scale).	1 (5), 2 (6), 3 (9), 4 (15), 5 (24), 6 (48), 7 (63), 8 (74), 9 (26), 10 (66)

2. English Proficiency and Employment

A significant 61.3% of respondents stated that their English proficiency helped them secure their current academic positions. However, 38.7% reported that English proficiency was not a factor, possibly due to non-English course offerings or hiring policies at the time of their employment.

3. English as a Job Requirement

Only 38.4% reported that English proficiency was a formal requirement for their positions, while the majority (61.6%) indicated that it was not. This suggests that many universities historically did not mandate English proficiency for employment but may now recognize its growing importance.

4. English as a Necessity in Academic Work

Most respondents strongly agreed (56.5%) or agreed (39.3%) that English is essential for their work, highlighting its importance in academic settings. Only a small fraction disagreed (3.3%) or strongly disagreed (0.9%).

5. English Proficiency as an Obstacle

More than half of the participants (181) acknowledged that a lack of English proficiency had been an obstacle in their academic work, while 155 respondents stated otherwise. This finding underscores the challenges faced by some academic staff in engaging with international materials and collaborations.

6. English and Performance in Other Academic Areas

A strong majority (62.8%) strongly agreed, and 34.5% agreed that English proficiency positively impacts other academic areas. Only a small percentage (2.4%) disagreed, indicating a near-universal recognition of English as a beneficial skill.

7. English Proficiency and Access to Materials

Many respondents (58.9% strongly agreed, 37.2% agreed) reported that their English skills helped them access academic materials that were unavailable in their mother tongue, further emphasizing the practical advantages of English proficiency.

8. Foreign Language Proficiency for University Staff

An overwhelming 69.9% strongly agreed and 30.1% agreed that university staff should be proficient in a language other than their mother tongue. This demonstrates a broad consensus on the value of multilingualism in academia.

9. English and Internationalization

The lack of English proficiency was perceived as a significant barrier to internationalization, with 56.3% stating it hinders development “a lot” and 38.1% considering it a “considerable” issue. Only 5.7% believed it had little impact.

10. Languages Used for Scientific Publications

English dominated as the preferred language for publishing scientific articles (72.9%), followed by Albanian (16.7%). Other languages such as German, Turkish, and mixed-language publications accounted for minor proportions.

11. Technology and English Proficiency

The majority strongly agreed (64.3%) or agreed (33.0%) that English proficiency is essential for using technology in academic settings, reinforcing the language’s role in modern education.

12. English and Professional Networking

A significant proportion of lecturers (67.0% strongly agreed, 29.2% agreed) believed that English had helped expand their professional networks, leading to additional career opportunities.

13. Self-Evaluation of English Abilities

Responses varied widely on a scale from 1 to 10, with the highest concentration around levels 6 to 8, indicating a generally moderate to high level of proficiency among the respondents.

14. Perceived Importance of English in Academic Work

Similar to the self-evaluation question, most responses clustered around higher ratings, with many respondents ranking English as highly important (scores of 7–10), further reinforcing its significance in academia.

## Open-ended questions - Summary section

The first one asked the lecturers, “Why is it important for you to improve your English language skills? (If you are already proficient, comment on why you think it is necessary for others to have some type of knowledge in another language).”

As for the responses, they were quite impressive and the most key ones were:

“1. No serious research can be done without using international publications, and the main language of those publications is English; 2. Nowadays, being bilingual is very important because it helps in your work; 3. For communication, literature, and lectures; 4. Because the English language mastery gives you more opportunities for everything an intellectual person needs, especially the academic staff; 5. English is the main language in the world used in fields such as: science, technology, and business; 6. To have access to more innovative literature; 7. To increase the quality of teaching; 8. To publish scientific works in English without having to pay for translations; 9. All science is in English, so proficiency in English is essential; 10. Mastery of English is essential at this time so that you can be up to date with the information and changes that are happening in the field of study. Given the fact that all the publications are being published in the English language; 11. As a lecturer, you must speak English in order to be as close as possible to developed countries; 12. English is the *Lingua franca* today; 13. Since I do not know English well, I suffer the great consequences of not being able to speak with foreign colleagues; 14. Today everything is in English; even university documents are translated into English; 15. If I knew English well, I would have a higher position; 16. Improving English language skills is a big step for all university employees; 17. Because English language proficiency is as important as the doctoral degree; 18. Knowledge of English is something elementary; it is a passport for us; 19. Indeed, English is more valuable than other diplomas and certificates. We translate research works mainly through translators who lose their value and quality; 20. It is important to improve English language skills because of scientific studies and international mobility; 21. It is perceived as a crucial support when materials; a supreme helper for presentations, participation in conferences, scientific projects, and publications; 22. The lecturers must improve their English skills so that they can have access to materials that are not translated into their mother tongue, but only in English; 23. The mastery of the English language is valuable because nowadays, all of the scientific articles are published in English, and the English language proficiency leads us to professional development and improves our academic performances.” 

And the second question was, “Because university staff continuously work with international institutions, does English language proficiency play a role in quality communication between the two?” The most key answers were:

“1. The main role, the better the communication, the more influential in fulfilling the requirements for reciprocity between institutions; 2. Increasing the quality of cooperation; 3. Development, quality improvement, exchange of experiences and knowledge; 4. An extremely important place in developing various areas of the University; 5. There is no quality communication without knowing English very well, and there is no international cooperation without mastering the English language; 6. The reputation of the university is different if there is quality communication; 7. Without communication, there is no cooperation, precisely in English, meetings with international colleagues take place mainly; 8. With quality communication we show our values as wise and hardworking people; 9. Applications, for example, for Erasmus+, Heras+, Horizon2020, are done in English; 10. English language proficiency plays an incomparable position in international collaborations and strengthens the reports of these international collaborations; most importantly, it plays a key role in the exchange of academic experiences with various universities around the world.” Only descriptive statistics (frequencies and percentages) were calculated to summarize responses. No inferential statistical analyses (e.g., correlation, regression, ANOVA) or cross-tabulations between demographic variables and proficiency levels were conducted, which limits the ability to identify predictors or group differences.

## Discussion

The findings reveal that participants in this survey considered their English language mastery to be at a medium level, with 65% rating their overall academic English as “good.” These findings highlight several systemic challenges for Kosovo’s higher education sector. First, while most lecturers self-report “good” or “very good” English proficiency, the absence of standardized descriptors such as CEFR levels limits the comparability and reliability of these self-assessments. Second, despite recognizing the importance of English for research, publication, and international collaboration, many institutions provide limited structured support for English development or English Medium Instruction (EMI) training. This aligns with international research showing uneven EMI preparedness ^15, 16^ and meta-analytic evidence that institutional support influences both content and language learning outcomes ^17^. Third, demographic trends in Kosovo, including declining student numbers and academic emigration, add urgency to strengthening English competence and internationalization strategies to sustain institutional viability. These challenges suggest that Kosovo’s universities must not only encourage individual English proficiency but also implement system-level policies, such as CEFR-aligned benchmarks, EMI training programs, and incentives for international collaboration, if they are to respond effectively to regional demographic and competitive pressures. 

For the next three questions on English language acknowledgment and usage in the public universities of Kosovo, English language proficiency was perceived as an important factor in securing academic positions, but when it came to job requirements, most of the lecturers answered negatively, since the majority of the lecturers have been lecturing in universities for a long time now and English language was not a requirement back then, and as for being considered as a necessity, 4.2% of the participants claimed that they do not see the English language usage that way. Furthermore, it is claimed that hardly any of the interviewed lecturers from Austria, Italy, or Poland seemed to have a clear understanding of what an adequate English language level could be and that some assumed that their selection had been predicated on whether they would have a doctorate from an Anglophone country, taught abroad, or were simply thought to speak English well by managers ^32^. A poll found that 83% of “informed observers” in 54 nations believed there were not enough competent lecturers in their country, even though the term “qualified” was left ambiguous, allowing it to be construed merely in terms of English language or qualification ^33^. Finally, it is obvious from a variety of study sources that lecturers in many academic fields believe their topic is less dependent on language. Nonetheless, considering the absence of lecturer English proficiency, it is obvious that the great majority of respondents believe that a high level of English language proficiency is necessary ^34^.

According to the participants’ answers to the next question, their response to the lack of English proficiency setting back the lecturers’ work can be seen as a demonstration of their English language proficiency lack in general, since recognizing the importance of English empowers lecturers to establish effective communication in their academic work. Inadequate resources certainly have an impact on the effectiveness of EMI policies ^35– 38^, and the shortcomings appeared to harm professors in this study. The EMI program was introduced by different foreign universities where superior facilities were expected. Due to the lack of fundamental resources, lecturers were put under unexpected pressure to change their teaching materials and techniques. The Internet, for example, may provide a wealth of English teaching tools ^39^.

The results also point out that lecturers at public universities in Kosovo perceived English language as a crucial support when materials in the lecturers’ mother tongue were unavailable. However, it is pointed out that the Danish language may be threatened by the increasing use of English as the language of the lecturers’ publications, and if Danish “ends up losing its domains,” that is, once it is no longer used in premium domains such as higher education, it will lose value and lead to a less developed vocabulary, which leads to more use of English ^27^.

Corresponding to the following questions on the development of internationalization and the academic staff’s being proficient in another language other than their mother tongue, the lecturers voted positively on these two questions, claiming that they see the need for other languages proficiency since English language proficiency itself and components such as mobility of students and the academic staff, teaching quality, and so on, play a vital role in the development of internationalization. Equivalent to this, many European countries and systems remain committed to teaching in the national language including the developed policies to encourage the use of the national languages alongside English ^40^.

According to the results, most of the lecturers claimed that the language they use for their scientific articles is the English language, whereas the rest of them claimed that they also publish their work in Albanian, German, Turkish, Bosnian, and other different foreign languages. According to this, it is stated that even though *Principia Mathematica* of Newton’s was written in Latin, yet, nowadays scientific articles are published mostly in English ^41^. A 2012 study published in the scientific journal Research Trends investigated publications gathered by Scopus, the world's biggest collection of peer-reviewed journals. To be eligible for inclusion in Scopus, a publication published in a language other than English should at the very least contain English abstracts. The study discovered that 80 percent of the more than 21,000 articles from 239 nations already in the database were written in English. The study focused on eight nations that generate a high number of scientific journals and discovered that the ratio of English to non-English articles had grown or stayed steady in all but one of them in the previous few years. Words such as oxygen or hydrogen sound like science. However, these terms originated from Russian, Greek, and French ^42^. Nowadays, though, even scientists and professors publish their work or their new discoveries in English. Some believe that Latin is the language of science, but when it comes to publications, they definitely use the English language.

The findings suggest that the majority of lecturers from public universities in Kosovo rated their English language abilities in general as “good”. In contrast, a large percentage of Turkey University lecturers evaluated their English positively and that more than 90 percent of those participating evaluated their English language level as “good” ^22^. Several studies concur in emphasizing the relevance of teaching staff’s linguistic and methodological expertise in ensuring the quality and proper execution of foreign programs. Furthermore, recent publications from several European authorities emphasize the importance of verifying these competencies ^43^. However, there is no agreement among Spanish universities on what level of English is required to teach topics in that language. Majority of Spanish universities required a B2 level to allow lecturers to enrol in EMI programs; 28 percent required a C1, and 34 percent had no prerequisite at all ^44^. Not only are there enormous differences across universities in terms of requirements, or absence thereof, as well as the level required, should that requirement exist, but there are also huge differences in the process of confirming ^29^.

After analyzing the effect of English language usage in public universities of Kosovo, the internationalization process, the English as a global language, all the results concluded that the utilization of the English language is seen as a dominant tool among the academic staff, that it presents higher education with opportunities and challenges, opportunities for developing education, and that it helps the academic staff get access to the world’s diverse knowledge. The internationalization of higher education also encourages cultural interactions, knowledge exchanges, and research collaboration. At the same time, it fosters understanding, mutual dependence, and friendships among universities and institutions worldwide.

## Conclusion

This study examined English language proficiency, perceptions, and competence among academic staff in Kosovo’s public universities. The majority of lecturers self-reported their English as “good” or “very good,” underscoring the language’s perceived importance for research, publication, and international collaboration. However, three key challenges emerged: (1) the absence of standardized proficiency benchmarks such as CEFR levels, which reduces comparability and precision; (2) limited institutional support and training for English Medium Instruction (EMI), despite its recognized importance; and (3) the urgent demographic pressures on Kosovo’s higher education system, including declining student numbers and academic emigration.

Based on these findings, we recommend that Kosovo’s universities and policymakers:

• Adopt CEFR-aligned benchmarks for staff language competence to ensure consistency and comparability.

• Provide structured EMI professional development, including targeted training and mentoring for academic staff.

• Integrate English proficiency and EMI support into internationalization strategies, using incentives such as mobility programs, collaborative research opportunities, and language development grants.

• Address demographic decline strategically by strengthening international appeal through English-friendly programs and support for faculty professional growth.

These actions can help Kosovo’s higher education institutions sustain quality, remain competitive, and respond proactively to demographic and global higher-education trends.

Finally, the study’s reliance on self-reported proficiency and descriptive statistics highlights the need for future research using standardized proficiency frameworks and more advanced quantitative analyses (e.g., correlations, regression) to explore how demographic and professional variables shape English competence.

## Ethics and consent

This study was conducted in accordance with the principles outlined in the Declaration of Helsinki. Ethical approval for this research study was obtained from the Scientific Committee of the University “Ukshin Hoti” Prizren on 20.09.2024. All participants provided written informed consent prior to participation.

## Data Availability

All the data regarding this study can only be used by the researchers in accordance with the consent form signed by the participant prior to participation. The data supporting this study are not publicly available due to ethical restrictions and participant confidentiality. Data access can be requested by contacting the lead author at sejdi.sejdiu@uni-prizren.com. Requests will be reviewed by the ethics committee at University “Ukshin Hoti” Prizren, and access may be granted under conditions that maintain participant confidentiality. However, the extended data supporting the findings of this study, including the questionnaire and consent form, are openly available on Zenodo under a **Creative Commons Attribution 4.0 International (CC BY 4.0)** licence at: https://doi.org/10.5281/zenodo.15828375 https://doi.org/10.5281/zenodo.16163429 ^45^
