# Supplementary material for: English language proficiency, perception and competence among the academic staff of public universities in Kosovo
Source: Open Res Eur. 2025 Oct 7;5:224. Originally published 2025 Aug 6. [Version 2] doi: 10.12688/openreseurope.19958.2 (PMC12754355; doi:10.12688/openreseurope.19958.2)
Supplement: Peer review history

# Reviewer report: Reviewer response for version 2

**DOI:** 10.21956/openreseurope.23270.r64773

**Published:** 2025-12-30

**Authors:** Sekoai Elliot Nkhi

**Affiliations:** 1 Communication Skills and Studies Unit, Centre for Academic Development, University of Botswana (Ringgold ID: 54547), Gaborone, South-East District, Botswana

The study sought to explore the English language proficiency, perception and competence among the academic staff of public universities in Kosovo. It shows the importance of English language not only in academic spaces but other spheres as well. However, the theoretical background of the phenomenon is not commensurate with the title of the study. This is because the key words depicted in the title are proficiency, perception and competence, but the background is centred around the globilisation of English and not the above mentioned key words. This misalignment therefore calls for a change in title of the study to reflect the background provided. I therefore suggests changing the title to ***'' Exploring the perception of English language proficiency and globilisation among academic staff in Kosovo's public universities''***. 

 Furthermore, the literature review provides an overview of English Language as a lingua franca and its impact as a language of instruction. The literature is thus well written and some gaps from previous studies are explored and the current study was able to fill those gaps especially in the context of Kosovo's higher education. However, there is no theoretical framework that informs the study. The researchers have to frame this study in order to help shape the readers understanding about the genesis of the phenomenon. 

 The methodology section presents the sample, the instruments, and data analysis in a coherent manner. The discussion is clearly presented and is linked to the objectives, and the researcher has demonstrated that the research objective that they set out to answer is addressed.

 Finally, the study can benefit from proper editing as there are many punctuation errors within the text. The language used is not a research based language. For instance, the following topic sentence should not be written like this, ***"In recent years, the English language is something you hear from politicians all across the world, say on the news*** ".

 The use of second person pronoun 'you' such as the one in this sentence *"... something **you** hear from politicians..*." must also be avoided in the  scholarship of research.

If applicable, is the statistical analysis and its interpretation appropriate?

Partly

Is the study design appropriate and is the work technically sound?

Partly

Is the work clearly and accurately presented and does it engage with the current literature?

Partly

Are the conclusions drawn adequately supported by the results?

Yes

Are sufficient details of methods and analysis provided to allow replication by others?

Yes

Are all the source data and materials underlying the results available?

Yes

Reviewer Expertise:

My areas of expertise are Applied Linguistics, Curriculum Development and Instruction, English Language Education, Second Language Acquisition, Translanguaging, Minority Language Media and Multilingualism. I was therefore able to assess all the aspects of the article.

I confirm that I have read this submission and believe that I have an appropriate level of expertise to confirm that it is of an acceptable scientific standard, however I have significant reservations, as outlined above.

# Reviewer report: Reviewer response for version 2

**DOI:** 10.21956/openreseurope.23270.r61770

**Published:** 2025-10-25

**Authors:** Nuhi Bllaca

**Affiliations:** 1 Department of English and American Studies, University of Vienna, Vienna, Vienna, Austria

I'm pleased to share that the authors of the article have addressed most of my concerns raised in the previous draft. However, there are still some minor issues that could further contribute to the quality of the paper. These issues mainly revolve around language and reference style use. For example, on p. 5, the authors use both the Vancouver reference style (with numbered citations) and also include the year of publication. In this case, they should have stuck to one consistent style, either APA or Vancouver (numerical).

 Here is the actual in-text citation, which was incorrectly referenced:

 "Recent developments in EMI scholarship have further enriched the field: for example, Macaro and Aizawa (2024) explore the interface between (15) EMI and EAP/ESP research agendas, Huang & Fang (2023)(16) investigate how EMI teachers integrate cultural dimensions in practice, and Lee et al. (2025)(17) provide meta-analytic evidence of EMI’s positive impact on both content learning and language proficiency. Meanwhile, Wang, Yuan, and De Costa (2025)(18)..."

 Next, this sentence is repeated twice: “The similarity of responses within these clusters during piloting, as well as the expert review process, provides reasonable assurance of the instrument’s reliability.”

 There are also cases where the authors do not provide necessary references. A typical example is the statement: “Common European Framework of Reference (CEFR).”

 Last but not least, a more engaging discussion section - one that compares and explains the study results in relation to previous research and further highlights the implications for the specific context in which the study was conducted - could strengthen the overall quality of the paper. Finally, the authors do not highlight the potential limitations of the study; and in cases where they do, these are not positioned appropriately within the text.

 I believe that by addressing the highlighted concerns, the authors will further improve the quality of their paper. However, these issues do not preclude me from granting my positive recommendation for the indexing of this article.

If applicable, is the statistical analysis and its interpretation appropriate?

Yes

Is the study design appropriate and is the work technically sound?

Yes

Is the work clearly and accurately presented and does it engage with the current literature?

Yes

Are the conclusions drawn adequately supported by the results?

Yes

Are sufficient details of methods and analysis provided to allow replication by others?

Partly

Are all the source data and materials underlying the results available?

No

Reviewer Expertise:

English for academic purposes; Peer interaction, Collaborative Writing; English language teaching both at pre-university and university contexts. Computer-mediated communications. Computer-assisted language learning. Task-based language teaching, etc.

I confirm that I have read this submission and believe that I have an appropriate level of expertise to confirm that it is of an acceptable scientific standard.

# Reviewer report: Reviewer response for version 2

**DOI:** 10.21956/openreseurope.23270.r61771

**Published:** 2025-10-23

**Authors:** Xhavit Rexhaj, Fidan Qerimi

**Affiliations:** 1 AAB College, Pristina, Kosovo (Serbia and Montenegro); 2 Economics, AAB College, Prishtina, Prishtina, Kosovo (Serbia and Montenegro)

Summary: In general,  this is a a significantly improved version of the article. Please, check and address few more comments (and refer to the initial comments) on the need for a more analytical approach in processing, analysis and discussion of the rich data obtained from the research.    

 1. To correct: Literature review starts with research questions!

 2. To correct: Authors refer to English Language competence as English ability!

 3. Quantitative analysis remains mainly descriptive with some comparisons with the results from other research. The data in the general part of the survey and the statements by the respondents could be more thoroughly analysed to compare responses of different groups of respondents (age, institutional ownership, filed of study etc.). The Article misses a lot in quality as a result of not addressing this analytical approach. Again, through cross-tabulation a lot of interesting results can be obtained (please read carefully our first comments).    

 4. There is still no genuine analysis and discussion of the results of the open-ended questions. 

 In the methodology section, Instrument and data analysis sub-section, the authors state that "Responses to the open-ended items were analyzed thematically: recurring patterns were identified and grouped into themes (e.g., access to international literature, career opportunities, communication with colleagues), and representative quotations were extracted to illustrate key points." I still do not see that analytical procedure applied in the respective section of the Article.

If applicable, is the statistical analysis and its interpretation appropriate?

No

Is the study design appropriate and is the work technically sound?

Partly

Is the work clearly and accurately presented and does it engage with the current literature?

Partly

Are the conclusions drawn adequately supported by the results?

Partly

Are sufficient details of methods and analysis provided to allow replication by others?

Partly

Are all the source data and materials underlying the results available?

Partly

Reviewer Expertise:

I, Xhavit Rexhaj, and my colleague Fidan Qerimi, are engaged in applied linguistics research using both qualitative and quantitative methods and instrument. We do not have any limitations to review this article.

We confirm that we have read this submission and believe that we have an appropriate level of expertise to confirm that it is of an acceptable scientific standard, however we have significant reservations, as outlined above.

# Reviewer report: Reviewer response for version 1

**DOI:** 10.21956/openreseurope.21589.r57892

**Published:** 2025-09-02

**Authors:** Xhavit Rexhaj, Fidan Qerimi

**Affiliations:** 1 AAB College, Pristina, Kosovo (Serbia and Montenegro); 2 Economics, AAB College, Prishtina, Prishtina, Kosovo (Serbia and Montenegro)

This article addresses a valuable topic in the Kosovo higher education. In fact, English language competence and improved internationalization may be one critical way out through a crisis of rapid decline in student numbers in Kosovo higher education institutions, which came as a result of recent demographic trends (steep decline in birth-rate and emigration of young people to western countries after recent EU visa liberalization).

 The introduction offers a valuable contextualization of language competence and internationalization in Kosovo, the expansion of staff mobility opportunities, the growth of online learning, and the emergence of artificial intelligence. This section also successfully situates the study within relevant developments in higher education and the broader socio-educational context of Kosovo. The link between language competence and internationalization is appropriately highlighted.

 Further below are presented key issues / comments that if adequately addressed would make this a notable scholarly contribution in the field.

 Literature Review:

 In several instances, in-text citations and quotations are not adequately integrated into the sentence structure – they should be introduced in a way that explicitly connects them to the argument being developed.

 Methodology:

 The study is referred to as a “case study,” yet the article does not provide sufficient evidence to substantiate this designation. The methodology section does not clearly define the object of the case study, nor does it outline additional methods (beyond the questionnaire) that would facilitate deeper analysis, triangulation, comparison, or verification of findings, common to case study research.

 The research is presented as employing a mixed-methods approach, combining quantitative and qualitative elements. However, the qualitative component appears limited. In their methodology section, the authors identify two elements of qualitative research: (a) reviewing reports and (b) including two open-ended questions in the questionnaire. First, it is questionable whether the reading and analysis of reports, without systematic qualitative analysis, constitutes a qualitative research method. Second, in the absence of interviews or focus group discussions—commonly employed in qualitative research—two open-ended questions may not be sufficient to substantiate a robust qualitative component.

 Even if the qualitative element were accepted as such, its presentation and analysis do not follow established qualitative analysis procedures (e.g., coding, identification of themes, categorization). Instead, the data are described rather than analyzed. This descriptive approach risks overlooking valuable insights that could have emerged from a thematic or category-based analysis.

 Regarding sampling, the authors should clarify whether the number of respondents is sufficient to support the generalizability of the findings. Additionally, further statistical analyses (e.g., factor analysis, regression analysis) would enhance the validity and reliability of the research instruments and results.

 Presentation and Analysis:

 The presentation of findings would benefit from tabular or graphical representations, particularly those allowing cross-tabulations or correlations between variables such as respondents’ institution, age, academic title, and field of study. Without such comparative analysis, potentially relevant patterns may remain undiscovered (for more on this see also the section of discussion below).

 In discussing faculty perceptions of their English language competence, it is unclear whether “good” levels of competence are consistently interpreted by respondents. It would be important to know whether respondents were provided with standardized descriptors of language proficiency (e.g., CEFR levels) to ensure reliability. Without such reference points, deeper analysis or follow-up interviews would be necessary to determine what “good” or “very good” actually signifies for the participants, but also for analysis of results.

 The authors could strengthen the analysis by comparing self-reported language competence data with results from other studies conducted in one or two higher education institutions in Kosovo.

 Discussion of Results:

 The discussion remains largely descriptive and does not fully engage in analytical interpretation of the findings. It would be beneficial to identify the key challenges faced by Kosovo’s higher education faculty and institutions with regard to language competence, its use in professional practice, and its role in career development.

 Analysis and Discussion:

 The analysis of the results in this paper is presented mainly in a descriptive form, which makes the presentation of the data clear and easy to follow. However, the analysis remains descriptive and falls short of using more advanced statistical methods, which would enable the extraction of deeper connections between variables and a more meaningful interpretation of the phenomenon. The results remain separate from each other, without being linked between variables such as age, academic title or field of study, which leaves room for potential differences between groups to not be identified. The discussion of the results remains mainly descriptive, since the results are presented without being accompanied by a more in-depth theoretical interpretation or direct comparisons with the literature. The use of inferential analyses, such as correlation, linear regression, factor analysis or ANOVA, would have helped to discover the factors that have the most influence and to build more reliable statistical connections. Also, comparing groups by academic title, experience, or field of study would have provided a more complete and understandable overview of the phenomenon studied. Including these elements would deepen the interpretation, increase the reliability of the findings, and contribute to a clearer positioning of the study within the existing scientific literature.

 Conclusions and Recommendations:

 This section requires greater alignment with the study’s findings. At present, English Medium Instruction (EMI) appears to resurface from the introduction and literature review rather than as a conclusion logically derived from the analysis. A more coherent link between the results, discussion, and conclusions is necessary.

 Language and Style:

 The article would benefit from a thorough linguistic revision to address typographical errors, improve vocabulary precision, and enhance syntactic clarity. A version of the article with this reviewer’s more concrete suggested edits is available.

If applicable, is the statistical analysis and its interpretation appropriate?

No

Is the study design appropriate and is the work technically sound?

Partly

Is the work clearly and accurately presented and does it engage with the current literature?

Partly

Are the conclusions drawn adequately supported by the results?

Partly

Are sufficient details of methods and analysis provided to allow replication by others?

Partly

Are all the source data and materials underlying the results available?

Partly

Reviewer Expertise:

I, Xhavit Rexhaj, and my colleague Fidan Qerimi, are engaged in applied linguistics research using both qualitative and quantitative methods and instrument. We do not have any limitations to review this article.

We confirm that we have read this submission and believe that we have an appropriate level of expertise to confirm that it is of an acceptable scientific standard, however we have significant reservations, as outlined above.

# Reviewer report: Reviewer response for version 1

**DOI:** 10.21956/openreseurope.21589.r57886

**Published:** 2025-09-02

**Authors:** Nuhi Bllaca

**Affiliations:** 1 Department of English and American Studies, University of Vienna, Vienna, Vienna, Austria

The study aimed to explore English language proficiency, perceptions, and competence among academic staff at public universities in Kosovo. Data were gathered from 366 participants across seven public universities in Kosovo. The paper makes a valuable contribution by addressing a gap in the literature on the role of English in the internationalization of higher education and its use as a medium of instruction (EMI). This is particularly significant in the Kosovar context, where no previous study has investigated this topic. The study also generated important findings regarding academic staff’s self-reported English proficiency and their views on the importance of English for academic work, research, and publication.

 However, before indexing, several improvements could strengthen quality of the paper:

 Introduction:

 The authors should clearly introduce the aims of the study in the first section (Introduction) and explicitly articulate the research gap relevant to the study’s purpose. They should also provide a clear statement on the expected contribution of the paper, particularly in the Kosovar context.

 Literature Review:

 Some of the research questions currently included in the Introduction should be moved to the Literature Review. In addition, the authors should incorporate more studies aligned with the topic or, where such studies are lacking, emphasize this absence. This would allow them to better highlight the research gap addressed by their study.

 Methodology:

 While the study uses a questionnaire, there is insufficient detail about its validity and reliability. The authors should provide more information about the design, piloting, and implementation of the questionnaire in order to assure readers of the robustness of the data-gathering instrument.

 Proofreading:

 The paper would benefit from careful proofreading to avoid minor inconsistencies or wording that could confuse readers.

 Minor Issues:

 A number of small issues have already been flagged in the text through comments and should be addressed prior to indexing.

If applicable, is the statistical analysis and its interpretation appropriate?

Yes

Is the study design appropriate and is the work technically sound?

Yes

Is the work clearly and accurately presented and does it engage with the current literature?

Yes

Are the conclusions drawn adequately supported by the results?

Yes

Are sufficient details of methods and analysis provided to allow replication by others?

Partly

Are all the source data and materials underlying the results available?

No

Reviewer Expertise:

English for academic purposes; Peer interaction, Collaborative Writing; English language teaching both at pre-university and university contexts. Computer-mediated communications. Computer-assisted language learning. Task-based language teaching, etc.

I confirm that I have read this submission and believe that I have an appropriate level of expertise to confirm that it is of an acceptable scientific standard, however I have significant reservations, as outlined above.
